# Alcohol use disorder diagnoses among commercially insured US adults from 2016 to 2023, by disability status and sex

**DOI:** 10.1016/j.dadr.2026.100461

**Published:** 2026-06-23

**Authors:** Rachel Sayko Adams, Sharon Reif, Yassir Mohamed, Maureen T. Stewart, Marc R. LaRochelle, Dennis Heaphy, John D. Corrigan, Fatema Shafie Khorassani, Jake R. Morgan

**Affiliations:** a Boston University School of Public Health, Department of Health Law, Policy and Management, Boston, MA, USA; b Brandeis University, Heller School for Social Policy and Management, Institute for Behavioral Health, Waltham, MA, USA; c Boston Medical Center and Boston University Chobanian & Avedisian School of Medicine, Boston, MA, USA; d Disability Policy Consortium, Boston, MA, USA; e The Ohio State University, Wexner Medical Center, Department of Physical Medicine and Rehabilitation, Columbus, OH, USA; f Boston University School of Public Health, Department of Biostatistics, Boston, MA, USA

**Keywords:** Alcohol use disorder, Disability, Sex, Commercial insurance

## Abstract

**Purpose:**

Epidemiologic survey studies suggest that people with disabilities are vulnerable to excessive alcohol use and alcohol use disorder (AUD), yet little is known about potential differences in clinical AUD diagnosis by disability status. Our objective was to examine trends in annual AUD diagnoses in the US from 2016 to 2023 among adults with commercial insurance, and to investigate potential differences by disability status/type, and by disability status/type and sex.

**Methods:**

Retrospective cross-sectional study using MarketScan commercial insurance claims from 2016 to 2023. Population-based sample of adults age 18–64 with commercial insurance across all 50 states. AUD diagnoses were identified with ICD-10 diagnosis codes. Age-adjusted annual AUD diagnosis rates per 10,000 population, stratified by disability status/type, and disability status/type and sex. Average annual percentage change of AUD rates over time.

**Results:**

The rate of AUD increased steadily from 85/10,000 persons in 2016 to 128/10,000 persons in 2023; average annual percentage change of 5.41% per year (CI 3.82% - 7.03%). Adults with disability had greater than threefold higher rates of AUD, and males and females with disabilities had a persistently higher rate of AUD compared to non-disabled males and females. Adults with serious mental illness and acquired brain injury were particularly likely to have AUD; rates were over 10 and 5 times higher than adults without these types of disabilities, respectively.

**Conclusions:**

Adults with disabilities had higher than three times the rate of AUD diagnoses compared to adults without disabilities. Interventions are needed inclusive of person-centered care for disabled people with comorbid AUD

## Highlights

• Age-adjusted annual AUD diagnosis rates increased from 2016 to 2023 in the US.
• Adults with disabilities had 3 times the rate of AUD than non-disabled adults.
• Males with disabilities had the highest rates of AUD.
• Women with disabilities had higher rates of AUD than non-disabled males or females.
• AUD treatment should address the unique needs of people with disabilities.

## 1 Introduction

Multiple studies have found that people with disabilities are less likely to drink alcohol compared to people without disabilities; yet, among those who consume alcohol, disabled adults appear to be at higher risk for excessive drinking than non-disabled adults (Adams et al., 2024, Reif et al., 2022). Population-level survey data from the 2018 Behavioral Risk Factor Surveillance Survey found that among people who currently drink alcohol, adults with disabilities had increased odds for past 30-day binge drinking (Adams et al., 2024). Similarly, the 2020 National Alcohol Survey found that adults with disabilities had more than double the days per year of engaging in high-intensity drinking (8 + drinks per day) compared to adults without disabilities (i.e., 19.7 versus 7.5 days) (Reif et al., 2022). Further, nationally-representative data from the 2018–2019 National Survey on Drug Use and Health found that disabled people had 80% higher odds of meeting criteria for past year alcohol use disorder (AUD) compared to non-disabled people (Aram et al., 2023). Despite these findings suggesting that people with disabilities have increased odds for meeting AUD criteria in self-reported survey data, it remains unknown if there are differences in clinical diagnosis of AUD by disability status, and if these trends change over time. These questions are critical as AUD is a chronic, relapsing condition which is associated with increased risk for alcohol-related morbidity and mortality (Committee on Review of Evidence on Alcohol and Health et al., 2025; Esser et al., 2024). Further, lower rates of clinical AUD diagnoses among adults with disabilities may suggest that the population is being overlooked in clinical assessment for AUD.

Significant additional gaps in the literature remain. Research on excessive alcohol use and AUD among people with disabilities has largely focused on subgroups of people with disability types (e.g., traumatic brain injury, spinal cord injuries, or intellectual and developmental disabilities) (Corrigan et al., 2021, Reif et al., 2023); there has not been systematic examination of differences in AUD diagnosis among the broader disability population. Further, while males traditionally consume more alcohol than females, gaps are narrowing for binge drinking and AUD (Grucza et al., 2018, Jager et al., 2021, Keyes et al., 2019, McCaul et al., 2019, White et al., 2015). However, it remains unknown if there are differences in AUD at the intersection of disability status/type and sex. To address these gaps, we evaluate trends in AUD diagnoses in the United States from 2016 to 2023 among adults with employer-based insurance, with a particular focus on potential differences by disability status and type, and disability status/type and sex.

## 2 Methods

### 2.1 Data source and sample

This repeated cross-sectional study leverages data from the Merative™ MarketScan® Commercial Database (MarketScan), spanning January 1, 2016, to December 31, 2023. These data contain individual-level, de-identified, commercial health insurance claims across the continuum of care (including inpatient, outpatient, retail pharmacy, carve-out behavioral healthcare) as well as enrollment data from large employers and health plans across the United States. We included US adults aged 18–64 with no missing unique enrollment identifiers, who had a minimum of nine months of continuous enrollment in a given year to establish a reliable window to identify the annual prevalence of AUD.

### 2.2 Measures

We defined the **outcome** of annual person-level AUD diagnosis as the presence of at least one AUD ICD−10 diagnosis code (F10.xx) on an inpatient or outpatient claim during the given year. The primary **exposure** of interest was overall disability status and specific type of disabling condition. Consistent with the medical model of disability and prior work to identify disability within healthcare claims research (Brown et al., 2021, Mitra et al., 2024, Thomas et al., 2023), disability was identified annually through ICD−10 diagnoses corresponding to specific types of disabling conditions: deaf or hard of hearing, blind or low-vision, acquired brain injuries, intellectual/developmental disabilities, severe mental illness, physical disabilities, and other neurological disorders (see Supplemental Appendix 1 for a full list of ICD−10 codes for all study variables, including disability types). *Disability status (yes/no)* was dichotomized as the presence of at least one disability type versus the absence of all included types within a given year. *Disability type (yes/no)* was defined by the individual presence or absence of each specific disability type (e.g., acquired brain injury versus no acquired brain injury). Thus, the dichotomous disability type indicators were not mutually exclusive. Sex was defined in the administrative billing claims (male, female).

### 2.3 Language

We aim to use non-stigmatizing language throughout our work, and we use both person-first (e.g., people with disabilities) and identity-first (e.g., disabled people) language to reflect the different preferences of people in the disability community (Dunn and Andrews, 2015).

### 2.4 Statistical analysis

We computed annual age-adjusted AUD rates per 10,000 population, which were stratified by disability status/type and sex. We determined AUD and disability status annually. For each calendar year, AUD status was determined by the presence of a diagnosis in that year. Similarly, disability status was determined by the presence of a qualifying diagnosis in that year. This approach improves cross year comparability by ensuring that each year focuses on individuals engaging in healthcare utilization for both AUD and (if applicable) a disabling condition in that given year. The annual AUD rate was calculated by dividing the total number of unique person-level AUD cases by the total number of enrollees with at least 9 months of continuous enrollment. We employed direct standardization, applying age-specific yearly AUD rates from our study population to the projected year 2000 U.S. standard population distribution across four age categories (18–24, 25–34, 35–44, and 45–64) (Centers for Disease Control and Prevention, 2025). This methodology yielded weighted average rates of current AUD diagnosis and the associated 95% confidence intervals from 2016 to 2023 across disability status/type and sex (Anderson and Rosenberg, 1998).

Overall trends in annual AUD rates were graphically presented, stratified by disability status/type and sex. We additionally present full stratification of rates by disability status, type, sex, and year in the Supplemental Appendix 2. To summarize temporal change in age-adjusted rates, we fit weighted log-linear regression models of the rate by calendar year for each subgroup, using inverse-variance weights. This process yielded average annual percent change (AAPC) for each subgroup, with 95% confidence intervals from model-based Wald standard errors. To evaluate the effect of COVID on our calculation of AAPC, we conducted a sensitivity analysis excluding the year 2020 from the AAPC calculation. Statistical analysis was conducted using SAS version 9.4. This study adheres to the EQUATOR Reporting Guidelines specific for cross-sectional studies, utilizing the Strengthening the Reporting of Observational Studies in Epidemiology guidelines. In accordance with 45 CFR §46.104(d)(4), Boston University's Institutional Review Board determined this study to be not human subjects research and exempt from review.

## 3 Results

Our sample included 42,086,684 adults age 18–64 with a similar proportion of males (48%) and females (52%). The proportion with a disability was relatively stable over time, ranging from 17.5% to 19.1% in a given year.

**Fig. 1:**
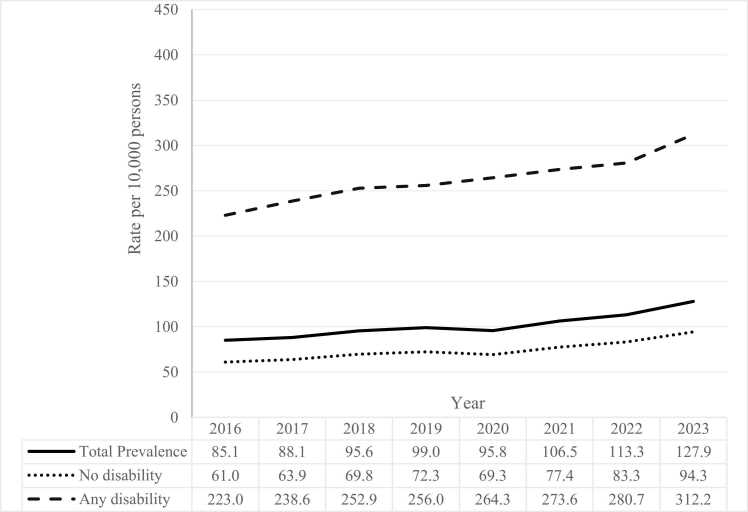
Annual Alcohol Use Disorder Rate 2016−2023 by disability status. Annual rate of AUD diagnosis per 10,000 persons. The solid line depicts total yearly AUD prevalence of the sample, the dashed line represents individuals with disabilities, and the dotted line represents individuals without disability*.*

**Fig. 2:**
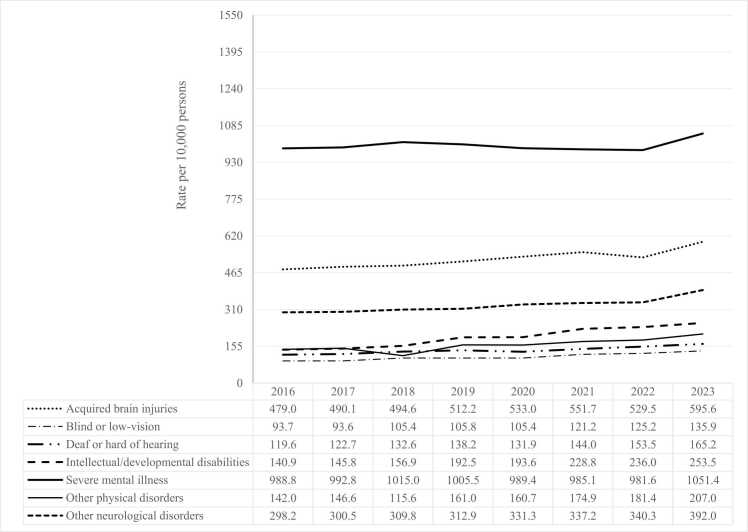
Annual Alcohol Use Disorder Rate by Disability Type 2016−2023. Annual prevalence of AUD diagnosis per 10,000 persons. The pattern of the line corresponds to the overall rate of AUD per 10,000 persons with documentation of that disability category by calendar year.

Age-adjusted rates of diagnosed AUD increased from 85.1 (95% Confidence Interval, 84.6–85.5) per 10,000 in 2016 to 127.9 (127.3–128.6) per 10,000 in 2023 (Fig. 1 and Supplemental Appendix 2). AUD rates increased for both adults with and without disabilities; however, disabled adults had more than threefold higher rates of AUD throughout the study period compared to non-disabled adults. Specifically, AUD diagnosis rates increased from 223.4 (221.3–225.5) per 10,000 in 2016 to 312.2 (309.4–315.0) per 10,000 in 2023 for adults with disabilities, compared to 61.0 (60.6–61.5) per 10,000 in 2016 to 94.3 (93.7–94.9) per 10,000 in 2023 for adults without disabilities. Throughout the study period, people with serious mental illness had the highest rates of AUD, followed by people with acquired brain injuries, and those with other neurologic disorders (Fig. 2).

**Fig. 3:**
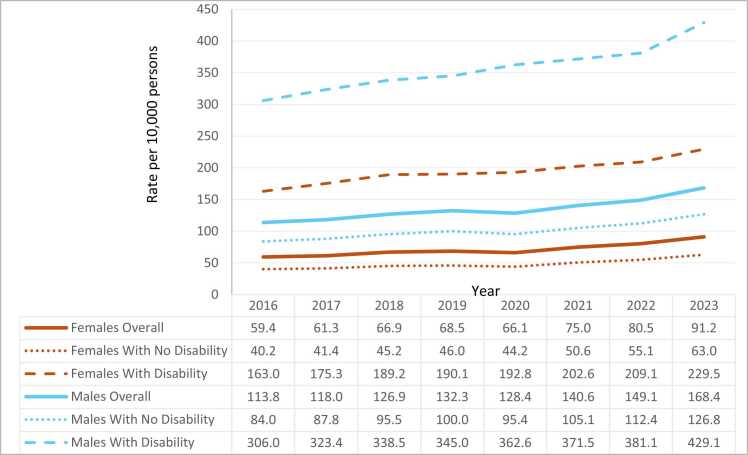
Annual Alcohol Use Disorder Rate Stratified by Sex and Disability Status 2016−2023. Annual rate of AUD diagnosis per 10,000 persons. Solid lines depicts total yearly prevalence of females (red) and males (blue), the dashed line represents individuals with disabilities, and the dotted line represents individuals without disability, colored for females (red) and males (blue) respectively.

When examining the intersection of disability status and sex, we found that males with disabilities, followed by females with disabilities, had the highest rates of AUD in all years, compared to both males and females without disabilities (Fig. 3). For example, the prevalence of AUD diagnosis in 2023 was 429.1 (424.1–434.2) per 10,000 for males with disabilities, and 229.5 (226.3–232.7) per 10,000 for females with disabilities, compared to 126.8 (125.8–127.8) per 10,000 for males without disabilities, and 63.0 (62.3–63.7) per 10,000 for females without disabilities. Both males and females with disabilities had over a threefold higher rate of AUD compared to males and females without disabilities respectively, with females with disabilities consistently closer to fourfold higher compared to females without disabilities. When examining the intersection of disability type with sex, males within each disability type had a higher AUD prevalence compared to females with the same type of disability (e.g., males with acquired brain injury versus females with acquired brain injury; Supplemental Appendix 2). The rank order of AUD prevalence by disability type was identical between males and females with disabilities in most years; the highest prevalence among those with serious mental illness and the lowest prevalence among those who are blind and low vision.

Overall, the rate of AUD rose an average of 5.41% per year (3.82–7.03, Table 1). While the rate of AUD was higher in disabled adults than in non-disabled adults, the average annual percent change did not differ materially between groups: 5.58% per year (4.06–7.12) for adults without disabilities vs 3.87% per year (2.89–4.90) for adults with disabilities (see Table 1, corresponding AAPC rows). All disability type subgroups experienced annual increases in AUD prevalence, ranging from an APPC of 2.10 (acquired brain injuries) to 9.63 (intellectual/developmental disabilities) except those with severe mental illness which was the only group with a non-significant AAPC, meaning the average yearly change was not statistically significantly different from zero. The subgroup with the highest AAPC was individuals with intellectual or developmental disabilities (9.63%, 7.52–11.92). While there was some modest variation in the point estimates of AAPC between males and females, the confidence intervals for each measure overlapped, implying the rate of change in AUD was similar by sex across disability subgroups. Our sensitivity analysis excluding the year 2020 from the calculation of the AAPC did not qualitatively change our results, full results are in the Supplemental Appendix 2.

**Table 1:** Average annual percent change of AUD rate by sample characteristic.

	**Total Sample**		**Female Sample**		**Male Sample**	
	Average Annual Percent Change	95% Confidence Interval	Average Annual Percent Change	95% Confidence Interval	Average Annual Percent Change	95% Confidence Interval
Overall	5.41	(3.82–7.03)	5.76	(3.92–7.67)	5.14	(3.70–6.64)
No Disability	5.58	(4.06–7.12)	6.03	(3.76–8.44)	5.30	(3.68–6.97)
Any Disability	3.87	(2.89–4.90)	3.84	(2.71–5.03)	4.12	(3.14–5.13)
Acquired brain injuries	2.10	(0.13–3.97)	3.03	(2.43–3.63)	2.31	(0.41–4.22)
Blind or low-vision	5.28	(3.86–6.69)	5.23	(3.95–6.63)	5.70	(3.54–8.06)
Deaf or hard of hearing	4.14	(2.49–5.75)	4.03	(2.10–6.09)	4.74	(3.15–6.40)
Intellectual/developmental disabilities	9.63	(7.52–11.92)	8.28	(4.43–12.99)	9.28	(6.27–13.05)
Severe mental illness	−0.03	(−0.82–0.73)	0.01	(−1.41–1.46)	0.91	(0.15–1.54)
Other physical conditions	4.87	(1.01–9.01)	4.60	(2.24–7.18)	4.84	(3.39–6.36)
Other neurological conditions	2.88	(1.49–4.24)	3.38	(1.84–4.98)	3.92	(1.72–6.18)

The average annual percentage year-over-year change across the sample 2016–2023 and the related confidence intervals

## 4 Discussion

To our knowledge, this is the first study to examine potential differences in diagnosed AUD by disability status using a commercial insurance population. Notably, we found significant variation in diagnosed AUD by disability status, with adults with a disability having greater than threefold higher rates of AUD than adults without disability. Our results are consistent with findings from the National Survey on Drug Use and Health which revealed that disabled adults were more likely to meet criteria for AUD based on self-reported items, compared to non-disabled adults (Aram et al., 2023). We also highlight that the overall rate of AUD increased steadily during the study period from a yearly prevalence of 85 per 10,000 persons in 2016 to 128 per 10,000 persons in 2023, representing an average annual percentage increase of 5.41% per year.

Our study also examined disability type in relation to trends in AUD diagnosis; attention to the heterogeneity of “disability” is critical to understanding the increasing rate of AUD among adults with disabilities. The dichotomy between serious mental illness and sensory disabilities (e.g., deaf or hard of hearing, blind or low-vision) is an example of this importance. We found adults with serious mental illness, which includes diagnoses of schizophrenia, bipolar disorder, and severe major depression, had by far the highest rate of comorbid AUD, double the rate of the second highest disability group (acquired brain injury) and ten times the rate of people without disabilities. While we did not find evidence that the rate of AUD among those with serious mental illness grew over the study period, given the high baseline prevalence rates, providers should be aware of this risk and be prepared to manage both conditions. Indeed, there has been established literature documenting the high rates of AUD among people with serious mental illness (Castillo-Carniglia et al., 2019). Alternatively, adults with IDD had lower than average rates of AUD among disabled people overall yet experienced a rapid increase in prevalence over our study period with an average AAPC of over 9% per year (95% CI, 7.52–11.92). This may represent a changing pattern of use or diagnosis that providers need to be aware of, particularly if providers for adults with sensory disabilities are less familiar with substance use issues compared to those who treat serious mental illness where the intersection with substance use has been persistently high. We also highlight that even the disability subgroups with the lowest rates of AUD (blind and low vision) had a higher AUD prevalence compared to people without disabilities.

Another innovation of our study was examining trends in AUD diagnosis rates at the intersection of disability and sex. We found that the rate of AUD was increasing just as fast among females as males, and that both males and females with disabilities had a persistently higher rates of AUD compared to males and females without disabilities. Similar to the overall disability status findings, both males and females with disabilities had over a threefold higher rate of AUD compared to males and females without disabilities respectively; however, females with disabilities had fourfold higher rates for most study years compared females without disabilities (Supplemental Appendix 2). This is important in the context of recent research showing the narrowing of the gap between AUD and other markers of heavy alcohol use between males and females (Grucza et al., 2018, Jager et al., 2021, Keyes et al., 2019, McCaul et al., 2019, White et al., 2015). Females with a disability had higher rates of AUD than males without a disability, underscoring the need for awareness of and access to treatment for this population. Compared to males, females are at greater risk of developing alcohol-related morbidity and mortality, including alcohol-related liver disease, cancer, and suicide (Agabio et al., 2016, Kezer et al., 2021, Lange et al., 2024, McCaul et al., 2019, The U.S. Surgeon General’s Advisory, 2025, White et al., 2018). Females are also less likely to engage in alcohol treatment than males (Gilbert et al., 2019, Levine et al., 2023). The high rate of AUD prevalence among females with disability indicates a substantial need for provider awareness regarding this overlap and for accessible and welcoming AUD treatment services for females with disability.

Taken together, prior evidence suggesting that disabled adults are more likely to progress to excessive drinking than non-disabled adults (Adams et al., 2024, Reif et al., 2022), combined with our findings revealing that disabled adults are more likely to be diagnosed with AUD than non-disabled adults, demonstrates the urgent need for research to understand why these differences exist. Prior research has demonstrated that people with disabilities experience worse mental health, social isolation, stigma, and social determinants of health (Krahn et al., 2015, Ledingham et al., 2022, Mitra et al., 2022, Reif et al., 2023). Primary data collection is needed to understand the unique experiences of people with lived experience with disability to understand if they are drinking due to social isolation, in attempts to fit in or in response to stigma experienced by disabled people, to manage anxiety or in response to their mental health, or for other reasons. Further, provider bias, stereotypes, or stigma may impact how providers talk about alcohol use with disabled patients and whether or not they screen for at-risk alcohol use (Reif et al., 2023, SAMHSA, 2012). A landmark study found that only a half of physicians in the US reported strongly welcoming patients with disabilities into their practice and only 41% felt very confident about their ability to provide the same quality care to their disabled patients as those without disabilities (Iezzoni et al., 2021).

A major strength of our study is the ability to examine a broad range of disability types longitudinally in a large dataset with over 40 million individuals across every state from 2016 to 2023. This allows us to accurately track trends in clinical AUD diagnosis at the intersection of disability and sex during our study period. However, our study has several important limitations. By relying on claims data we are limited to what is documented during a medical visit. In any given year, the only individuals identified with AUD or with a disability are those with a relevant documented ICD−10 diagnosis code in that year. This undercounts the “true” number of individuals with an AUD or disability who may not seek care for that condition, or have that condition recognized by a provider, in the given calendar year. We know AUD is vastly underdiagnosed for a variety of reasons including stigma (Rundle et al., 2021). Our results likely capture the most clinically significant, and therefore documented AUD; thus, may not reflect changes in the prevalence of AUD that is not documented in a medical setting. If individuals with AUD sought care through private pay settings this would not be captured in the data; however, because the commercial plans are self-insured, any carve-out services for mental health or substance use treatments that are covered were captured.

Due to persistent underdiagnosis of AUD, and consistent with prior research (Dabbas et al., 2026), we do not rely on ICD−10 codes to attempt to create a hierarchy of AUD severity. If individuals with more severe disease are more likely to be diagnosed, this implies we are likely disproportionally capturing individuals with higher AUD severity. This also means that the changes in prevalence identified here may reflect a change in diagnostic patterns of AUD. While we do not have reason to believe that diagnostic patterns have undergone large changes over our study period, or would be different among our subgroups, future research should seek to understand the relationship between diagnostic patterns, including whether certain providers or settings are more likely to result in an AUD diagnosis. It is important to note that adults with disabilities tend to have poorer overall health and more underlying medical conditions, and thus may interact with the medical system more frequently (Adams et al., 2024, Krahn et al., 2015), providing more opportunities for diagnosis of AUD. However, we do not suspect that this would explain the more than threefold higher rates of AUD that we saw throughout the study period for disabled adults compared to non-disabled adults. We note that relying on claims data to identify disability is consistent with the medical model of disability and does not capture functional disability or severity (Mitra et al., 2022). However, within claims it is an important proxy for providers’ identification/diagnosis of known disabilities. Lastly, our study may not be generalizable to adults with non-commercial health insurance or uninsured populations. Yet, our study generalizes to the approximately two-thirds of people in the US who have commercial insurance (Bunch and Katema, 2025). Substance use disorder is disproportionally higher among people with Medicaid insurance (Substance Abuse and Mental Health Services Administration, 2025). Similar studies should be conducted with Medicaid and other insurance populations to examine if there are similar or divergent trends in AUD diagnosis by disability status.

## 5 Conclusions

Both males and females with medically documented disabilities are at high and increasing risk for AUD, compared to adults without disabilities. Importantly, this higher risk for AUD is found across all disability types, and for both males and females with disabilities. It is important for providers to know that adults with serious mental illness, acquired brain injuries, and other neurologic disorders are at particularly elevated risk. Efforts are needed to ensure that providers are aware of this risk, and that they are prepared to screen for AUD and offer evidence-based treatment services. People with disabilities may have unique needs and require person-centered alcohol treatment protocols that are designed specifically to accommodate their conditions. More research is needed on how best to tailor treatment services and successfully engage people with various types of disabilities (Iezzoni et al., 2021), including adults with acquired brain injury, neurologic conditions and other disabilities, as well as AUD treatment options that consider women-specific treatment options.

## CRediT authorship contribution statement

**Rachel Sayko Adams:** Writing – original draft, Supervision, Project administration, Methodology, Investigation, Funding acquisition, Conceptualization. **Sharon Reif:** Writing – original draft, Project administration, Investigation, Funding acquisition, Conceptualization. **Yassir Mohamed:** Writing – original draft, Visualization, Investigation, Formal analysis, Data curation. **Maureen T. Stewart:** Writing – original draft, Funding acquisition, Conceptualization. **Marc R. LaRochelle:** Writing – original draft, Conceptualization. **Dennis Heaphy:** Writing – original draft, Investigation. **John D. Corrigan:** Writing – original draft, Investigation, Conceptualization. **Fatema Shafie Khorassani:** Writing – original draft, Methodology, Investigation. **Jake R. Morgan:** Writing – original draft, Visualization, Supervision, Project administration, Methodology, Investigation, Funding acquisition, Formal analysis, Data curation, Conceptualization.

## Funding

This work was supported by the 10.13039/100000027National Institute on Alcohol Abuse and Alcoholism (R01AA031236) and the 10.13039/100000026National Institute on Drug Abuse (P30DA040500). The funding source had no role in the design and conduct of the study; collection, management, analysis, and interpretation of the data; preparation, review, or approval of the manuscript; and decision to submit the manuscript for publication. Certain data were supplied by Merative as part of one or more MarketScan Research Databases. Any analysis, interpretation, or conclusion based on these data is solely that of the authors and not Merative.

## Declaration of Competing Interest

The authors declare that they have no known competing financial interests or personal relationships that could have appeared to influence the work reported in this paper.

## Footnotes

Appendix A Supplementary data associated with this article can be found in the online version at doi:10.1016/j.dadr.2026.100461.
