# Supplementary material for: Alcohol use disorder diagnoses among commercially insured US adults from 2016 to 2023, by disability status and sex
Source: Drug Alcohol Depend Rep. 2026 Jun 23;20:100461. doi: 10.1016/j.dadr.2026.100461 (PMC13333359; doi:10.1016/j.dadr.2026.100461)
Supplement: Supplementary file 1 — Supplementary material [file mmc1.docx]

**Supplemental Appendix 1 –ICD-10 diagnostic codes for disability types**

| **Disability Type** | **ICD10 Codes** |
| --- | --- |
| Blind or low-vision | H31, H34, H35, H36, H44, H46, H47, H54, Q11, Q13 |
| Deaf or hard of hearing | H90, H91, Q16 |
| Acquired brain injuries | F0781, S06, S071, G81, G91, G931, I60, I61, I63, I64, I69 |
| Intellectual/  developmental disabilities | F70, F71, F72, F73, F78, F79, F84, F88, Q86, Q90, Q91, Q92, Q93, Q992, Q998 |
| Severe mental illness | F20, F25, F22, F24, F28, F29, F30, F310, F31, F310, F311, F3110, F3111, F3112, F3113, F312, F313, F3130, F3131, F3132, F314, F315, F316, F3160, F3161, F3162, F3163, F3164, F317, F3170, F3171, F3172, F3173, F3174, F3175, F3176, F3177, F3178, F3181, F3189, F319, F323, F332, F333 |
| Other physical conditions | E22, E230, E343, E84, G32, G54, G55, G57, G58, G59, G60, G61, G62, G63, G70, G82, G83, G834, G901, G95, M05, M06, M15, M16, M17, M18, M19, M218, M224, M23, M35, M42, M45, M46, M47, M50, M51, M80, M86, M87, M91, M92, M93, Q019, Q02, Q03, Q04, Q05, Q06, Q07, Q66, Q67, Q70, Q71, Q72, Q73, Q74, Q75, Q76, Q77, Q78, Q79, S14, S24, S34, S48, S58, S68, S77, S87, S97, T04, S78, S88, S98, T05, T06, Z89 |
| Other neurological conditions | B91, F00, F01, F02, F03, G10, G11, G12, G20, G21, G23, G24, G25, G30, G31, G35, G36, G37, G40, G71, G72, G80, G90 |
