# Supplementary material for: Alcohol use disorder diagnoses among commercially insured US adults from 2016 to 2023, by disability status and sex
Source: Drug Alcohol Depend Rep. 2026 Jun 23;20:100461. doi: 10.1016/j.dadr.2026.100461 (PMC13333359; doi:10.1016/j.dadr.2026.100461)
Supplement: Supplementary file 2 — Supplementary material [file mmc2.docx]

**SUPPLEMENTAL APPENDIX 2**

| **Supplemental Table:** **Age-adjusted AUD rates by year, disability, and sex** | | | | | | | | |
| --- | --- | --- | --- | --- | --- | --- | --- | --- |
|  | Total | | Females | | Males | |  |  |
|  | Rate AUD / 10,000 | 95% CI | Rate AUD / 10,000 | 95% CI | Rate AUD / 10,000 | 95% CI | |  |
|  | **2016** | | | | | | | |
| Total | 85.1 | 84.6 - 85.5 | 59.4 | 58.9 - 59.9 | 113.8 | 113.1 - 114.6 | |  |
| No disability | 61.0 | 60.6 - 61.5 | 39.8 | 39.3 - 40.3 | 83.6 | 82.9 - 84.3 | |  |
| Any disability | 223.4 | 221.3 - 225.5 | 162.9 | 160.5 - 165.3 | 306.2 | 302.4 - 309.9 | |  |
| ABI | 479.1 | 468.7 - 489.4 | 294.3 | 283.8 - 304.8 | 745.6 | 724.6 - 766.7 | |  |
| Blind | 93.7 | 89.1 - 98.2 | 64.7 | 59.7 - 69.7 | 135.9 | 127.3 - 144.5 | |  |
| DHH | 119.6 | 114.1 - 125.1 | 83.3 | 76.9 - 89.8 | 159.7 | 150.4 - 168.9 | |  |
| IDD | 140.9 | 120.4 - 161.4 | 102.7 | 76.1 - 129.3 | 183.9 | 151.0 - 216.8 | |  |
| SMI | 988.8 | 976.0 - 1001.7 | 762.6 | 748.4 - 776.7 | 1391.5 | 1365.9 - 1417.0 | |  |
| Physical | 141.8 | 139.8 - 143.9 | 98.7 | 96.4 - 101.0 | 203.2 | 199.4 - 207.0 | |  |
| Other | 298.2 | 291.4 - 304.9 | 199.7 | 192.4 - 206.9 | 453.7 | 440.6 - 466.9 | |  |
|  | **2017** | | | | | | | |
| Total | 88.1 | 87.7 - 88.6 | 61.3 | 60.7 - 61.8 | 118.0 | 117.3 - 118.8 | |  |
| No disability | 63.9 | 63.5 - 64.3 | 41.4 | 40.9 - 41.9 | 87.8 | 87.1 - 88.6 | |  |
| Any disability | 238.6 | 236.3 - 240.9 | 175.3 | 172.6 - 177.9 | 323.4 | 319.3 - 327.5 | |  |
| ABI | 490.1 | 476.4 - 503.9 | 303.5 | 288.9 - 318.0 | 716.8 | 691.4 - 742.1 | |  |
| Blind | 93.6 | 89.0 - 98.1 | 65.1 | 60.1 - 70.2 | 134.9 | 126.3 - 143.5 | |  |
| DHH | 122.7 | 117.1 - 128.3 | 84.1 | 77.7 - 90.5 | 165.0 | 155.5 - 174.5 | |  |
| IDD | 145.8 | 124.9 - 166.6 | 125.5 | 136.9 - 197.9 | 167.4 | 95.6 - 155.4 | |  |
| SMI | 992.8 | 979.9 - 1005.6 | 755.9 | 741.9 - 769.8 | 1420.5 | 741.9 - 769.8 | |  |
| Physical | 146.6 | 144.3 - 148.9 | 101.7 | 99.1 - 104.3 | 208.4 | 204.3 - 212.6 | |  |
| Other | 300.5 | 293.6 - 307.4 | 204.3 | 196.8 - 211.9 | 448.2 | 434.9 - 461.4 | |  |
|  | **2018** | | | | | | | |
| Total | 95.6 | 95.1 - 96.0 | 66.9 | 66.4 - 67.5 | 126.9 | 126.1 - 127.7 | |  |
| No disability | 69.8 | 69.3 - 70.3 | 45.2 | 44.7 - 45.7 | 95.5 | 94.7 - 96.2 | |  |
| Any disability | 252.9 | 250.6 - 255.2 | 189.2 | 186.5 - 191.9 | 338.5 | 334.4 - 342.6 | |  |
| ABI | 494.6 | 481.2 - 508.1 | 321.1 | 306.6 - 335.5 | 707.2 | 682.7 - 731.7 | |  |
| Blind | 105.4 | 100.7 - 110.1 | 70.5 | 65.5 - 75.6 | 155.6 | 146.4 - 164.8 | |  |
| DHH | 132.6 | 127.0 - 138.3 | 93.3 | 86.7 - 99.9 | 176.2 | 166.7 - 185.7 | |  |
| IDD | 156.9 | 134.5 - 179.4 | 100.5 | 73.7 - 127.4 | 201.5 | 166.9 - 236.1 | |  |
| SMI | 1015.0 | 1002.4 - 1027.6 | 774.2 | 760.5 - 787.9 | 1447.2 | 1421.9 - 1472.5 | |  |
| Physical | 115.6 | 156.2 - 161.0 | 112.8 | 110.1 - 115.6 | 221.5 | 217.3 - 225.7 | |  |
| Other | 309.8 | 302.8 - 316.7 | 210.4 | 202.8 - 217.9 | 464.4 | 450.9 - 477.8 | |  |
|  | **2019** | | | | | | | |
| Total | 99.0 | 98.5 - 99.5 | 68.5 | 67.9 - 69.1 | 132.3 | 131.4 - 133.1 | |  |
| No disability | 72.3 | 71.8 - 72.8 | 46.0 | 45.5 - 46.6 | 99.6 | 98.8 - 100.4 | |  |
| Any disability | 256.0 | 253.6 - 258.4 | 190.1 | 187.3 - 192.9 | 344.6 | 340.5 - 348.8 | |  |
| ABI | 512.2 | 498.1 - 526.3 | 332.5 | 317.4 - 347.6 | 737.8 | 711.8 - 763.9 | |  |
| Blind | 105.8 | 100.9 - 110.6 | 72.7 | 67.3 - 77.9 | 152.8 | 143.8 - 161.8 | |  |
| DHH | 138.2 | 132.3 - 144.1 | 94.1 | 87.3 - 100.9 | 186.7 | 176.8 - 196.6 | |  |
| IDD | 192.5 | 167.5 - 217.6 | 138.3 | 105.8 - 170.8 | 233.8 | 196.5 - 271.1 | |  |
| SMI | 1005.5 | 992.8 - 1018.2 | 764.7 | 750.9 - 778.5 | 1441.2 | 1415.6 - 1466.8 | |  |
| Physical | 161.0 | 158.5 - 163.4 | 113.4 | 110.6 - 116.2 | 226.6 | 222.3 - 231.0 | |  |
| Other | 312.9 | 305.8 - 320.2 | 216.9 | 209.1 - 224.8 | 459.4 | 445.7 - 473.1 | |  |
|  | **2020** | | | | | | | |
| Total | 95.8 | 95.3 - 96.3 | 66.1 | 65.5 - 66.7 | 128.4 | 127.5 - 129.2 | |  |
| No disability | 69.3 | 68.8 - 69.7 | 44.2 | 43.7 - 44.8 | 95.4 | 94.6 - 96.2 | |  |
| Any disability | 264.3 | 261.8 - 266.9 | 192.8 | 189.9 - 195.7 | 362.6 | 358.1 - 367.2 | |  |
| ABI | 533.0 | 517.4 - 548.7 | 344.8 | 328.0 - 361.5 | 764.1 | 735.5 - 792.7 | |  |
| Blind | 105.4 | 100.2 - 110.5 | 71.3 | 65.7 - 76.8 | 154.9 | 145.0 - 164.7 | |  |
| DHH | 131.9 | 125.5 - 138.2 | 85.2 | 78.1 - 92.2 | 183.8 | 172.9 - 194.7 | |  |
| IDD | 193.6 | 168.1 - 219.1 | 139.0 | 106.8 - 171.3 | 232.9 | 195.4 - 270.5 | |  |
| SMI | 989.4 | 976.7 - 1002.1 | 735.0 | 721.5 - 748.6 | 1459.0 | 1432.9 - 1485.0 | |  |
| Physical | 160.7 | 158.1 - 163.2 | 109.4 | 106.6 - 112.2 | 232.4 | 227.7 - 237.0 | |  |
| Other | 331.3 | 323.7 - 338.9 | 218.5 | 210.4 - 226.6 | 507.6 | 492.7 - 522.6 | |  |
|  | **2021** | | | | | | | |
| Total | 106.5 | 105.9 - 107.0 | 75.0 | 74.4 - 75.7 | 140.6 | 139.7 - 141.5 | |  |
| No disability | 77.4 | 76.9 - 77.9 | 50.6 | 50.0 - 51.2 | 105.1 | 104.2 - 105.9 | |  |
| Any disability | 273.6 | 271.0 - 276.2 | 202.6 | 199.6 - 205.5 | 371.5 | 366.9 - 376.1 | |  |
| ABI | 551.7 | 536.1 - 567.4 | 354.6 | 337.9 - 371.2 | 796.2 | 767.4 - 825.1 | |  |
| Blind | 121.2 | 115.7 - 126.7 | 82.7 | 76.7 - 88.8 | 175.6 | 165.2 - 185.9 | |  |
| DHH | 144.0 | 137.7 - 150.4 | 100.1 | 92.7 - 107.5 | 192.9 | 182.1 - 203.6 | |  |
| IDD | 228.8 | 201.3 - 256.3 | 161.4 | 127.3 - 195.6 | 279.7 | 238.6 - 320.8 | |  |
| SMI | 985.1 | 972.5 - 997.7 | 738.7 | 725.3 - 752.2 | 1452.6 | 1426.6 - 1478.6 | |  |
| Physical | 174.9 | 172.3 - 177.6 | 121.0 | 118.0 - 123.9 | 249.9 | 245.1 - 254.7 | |  |
| Other | 337.2 | 329.5 - 344.9 | 224.3 | 216.1 - 232.4 | 515.1 | 499.9 - 530.2 | |  |
|  | **2022** | | | | | | | |
| Total | 113.3 | 112.7 - 113.9 | 80.5 | 79.9 - 81.2 | 149.1 | 148.2 - 150.1 | |  |
| No disability | 83.3 | 82.7 - 83.8 | 55.1 | 54.5 - 55.7 | 112.4 | 111.5 - 113.3 | |  |
| Any disability | 280.7 | 278.1 - 283.3 | 209.1 | 206.1 - 212.1 | 381.1 | 376.4 - 385.8 | |  |
| ABI | 529.5 | 514.2 - 544.7 | 351.4 | 335.0 - 367.8 | 757.2 | 728.9 - 785.6 | |  |
| Blind | 125.2 | 119.8 - 130.7 | 86.0 | 80.0 - 91.9 | 181.9 | 171.4 - 192.3 | |  |
| DHH | 153.5 | 147.0 - 160.1 | 107.1 | 99.5 - 114.8 | 205.9 | 194.9 - 216.8 | |  |
| IDD | 236.0 | 208.7 - 263.3 | 142.6 | 111.6 - 173.7 | 306.6 | 264.6 - 348.5 | |  |
| SMI | 981.6 | 969.1 - 994.2 | 744.5 | 730.9 - 757.9 | 1429.1 | 1403.4 - 1454.8 | |  |
| Physical | 181.4 | 178.7 - 184.2 | 126.6 | 123.6 - 129.7 | 259.2 | 254.2 - 264.2 | |  |
| Other | 340.3 | 332.6 - 348.1 | 229.2 | 220.9 - 237.4 | 519.2 | 503.9 - 534.6 | |  |
|  | **2023** | | | | | | | |
| Total | 127.9 | 127.3 - 128.6 | 91.2 | 90.4 - 91.9 | 168.4 | 167.4 - 169.5 | |  |
| No disability | 94.3 | 93.7 - 94.9 | 63.0 | 62.3 - 63.7 | 126.8 | 125.8 - 127.8 | |  |
| Any disability | 312.2 | 309.4 - 315.0 | 229.5 | 226.3 - 232.7 | 429.1 | 424.1 - 434.2 | |  |
| ABI | 595.6 | 579.1 - 612.0 | 375.5 | 358.0 - 392.9 | 867.8 | 837.5 - 898.1 | |  |
| Blind | 135.9 | 130.2 - 141.5 | 91.0 | 85.0 - 97.1 | 201.1 | 190.2 - 212.0 | |  |
| DHH | 165.2 | 158.4 - 172.0 | 110.3 | 102.6 - 118.0 | 228.0 | 216.2 - 239.8 | |  |
| IDD | 253.5 | 226.3 - 280.6 | 193.3 | 159.0 - 227.6 | 303.1 | 262.3 - 343.9 | |  |
| SMI | 1051.4 | 1038.5 - 1064.2 | 789.3 | 775.5 - 803.1 | 1543.3 | 1516.8 - 1569.7 | |  |
| Physical | 207.0 | 204.0 - 210.0 | 145.8 | 142.4 - 149.1 | 294.8 | 289.4 - 300.3 | |  |
| Other | 392.0 | 383.7 - 400.4 | 263.0 | 254.4 - 271.7 | 607.1 | 590.1 - 624.0 | |  |

**Abbreviations:** DHH - deaf or hard of hearing, Blind - blind or low-vision; ABI - acquired brain injuries; IDD - intellectual/developmental disabilities; SMI - severe mental illness; Physical - physical disabilities; and Other - other neurological disorders

**Supplemental Table: Table 1: Average annual percent change of AUD rate by sample characteristic excluding 2020**

|  | **Total Sample** | | | **Female Sample** | | | **Male Sample** | | |
| --- | --- | --- | --- | --- | --- | --- | --- | --- | --- |
|  | Average Annual Percent Change | 95% Confidence Interval | P-Value | Average Annual Percent Change | 95% Confidence Interval | P-Value | Average Annual Percent Change | 95% Confidence Interval | P-Value |
| Overall | 5.52 | (3.93 - 7.13) | <0.01 | 5.86 | (3.77 - 8.11) | <0.01 | 5.22 | (4.28 - 6.18) | <0.01 |
| No Disability | 5.72 | (4.34 - 7.10) | <0.01 | 3.87 | (2.78 - 5.02) | <0.01 | 4.13 | (2.81 - 5.48) | <0.01 |
| Any Disability | 3.88 | (2.69 - 5.14) | <0.01 | 6.14 | (3.91 - 8.55) | <0.01 | 5.38 | (4.08 - 6.76) | <0.01 |
| Acquired brain injuries | 2.06 | (-0.62 - 4.59) | 0.12 | 3.01 | (2.40 - 3.60) | <0.01 | 2.32 | (-0.51 - 5.16) | 0.12 |
| Blind or low-vision | 5.44 | (3.82 - 7.02) | <0.01 | 5.28 | (4.77 - 5.84) | <0.01 | 5.75 | (3.73 - 7.98) | <0.01 |
| Deaf or hard of hearing | 4.28 | (2.47 - 6.03) | <0.01 | 4.14 | (3.46 - 4.87) | <0.01 | 4.77 | (3.55 - 6.09) | <0.01 |
| Intellectual/developmental disabilities | 9.66 | (6.66 - 13.11) | <0.01 | 8.27 | (4.58 - 12.87) | <0.01 | 9.28 | (7.16 - 11.85) | <0.01 |
| Severe mental illness | -0.01 | (-1.16 - 1.06) | 0.94 | 0.04 | (-1.00 - 1.11) | 0.89 | 0.91 | (0.11 - 1.55) | 0.02 |
| Other physical conditions | 4.93 | (0.54 - 9.30) | 0.03 | 4.69 | (2.33 - 7.23) | <0.01 | 4.89 | (3.59 - 6.22) | <0.01 |
| Other neurological conditions | 2.85 | (1.10 - 4.57) | <0.01 | 3.40 | (2.18 - 4.71) | <0.01 | 3.91 | (0.66 - 7.27) | 0.02 |
